# Distribution of subpopulations of dendritic cells in peripheral blood of patients treated with exogenous thyrotropin

**DOI:** 10.1186/1756-6614-5-18

**Published:** 2012-11-30

**Authors:** Mariusz Stasiołek, Zbigniew Adamczewski, Bartosz Puła, Kinga Krawczyk-Rusiecka, Arkadiusz Zygmunt, Magdalena Borowiecka, Piotr Dzięgiel, Andrzej Lewiński

**Affiliations:** 1 Department of Endocrinology and Metabolic Diseases, Polish Mother’s Memorial Hospital - Research Institute, Rzgowska Str. 281/289, 93-338, Lodz, Poland; 2 Department of Endocrinology and Metabolic Diseases, Medical University of Lodz, Lodz, Poland; 3 Department of Histology and Embryology, Medical University of Wroclaw, Wroclaw, Poland; 4 Department of Physiotherapy, Wroclaw University School of Physical Education, Wroclaw, Poland

**Keywords:** Thyrotropin, Dendritic cells, Immunoregulation

## Abstract

**Background:**

Dendritic cells (DCs) play a major role as regulators of inflammatory events associated with thyroid pathology. The immunoregulatory function of DCs depends strongly on their subtype, as well as maturation and activation status. Numerous hormonal factors modulate the immune properties of DCs, however, little is known about effects exerted by the hypothalamus-pituitary-thyroid-axis. Recently, we have shown a direct regulatory influence of thyroid hormones (TH) on human DCs function. The aim of the present study was to analyze the effect of systemically administered thyrotropin (TSH) on human blood DCs *ex vivo*.

**Methods:**

Blood samples for the cytometric analysis of peripheral blood plasmacytoid and myeloid DCs subtypes were collected from patients subjected to total thyroidectomy because of differentiated thyroid carcinoma at 2 time points: (i) directly before the commencement of TSH administration and (ii) 5 days after first TSH injection. The whole blood quantitative and phenotypic analysis of plasmacytoid and myeloid DCs subtypes was performed by flow cytometry.

**Results:**

Administration of TSH did not influence the percentage of plasmacytoid DCs in peripheral blood of study participants. Also the percentage of the two main myeloid DCs subpopulations – CD1c/BDCA1+ DCs and CD141/BDCA3+ DCs did not change significantly. TSH administration had no effect on the surface expression of CD86 – one of the major costimulatory molecules – neither in the whole peripheral blood mononuclear cell (PBMC) fraction nor in particular DCs subtypes.

**Conclusions:**

In the present study, we demonstrated no influence of systemic TSH administration on human peripheral blood DCs subtypes. These results are in accordance with our previous work suggesting the direct effect of TH on human DCs *ex vivo*.

## Background

A complex network of immune-endocrine interactions involving numerous cell types, humoral immune mediators and different hormonal systems participate in many of the physiological processes and, when disbalanced, also in pathological mechanisms leading to various disorders, including autoimmunity, malignancies, atherosclerosis and infertility [1-3]. The influence of immune system on thyroid function has extensively been investigated in several experimental models of autoimmune thyroid disease, as well as in multiple cases of human thyroid tumors. Increasing body of evidence underlines the pivotal role of dendritic cells (DCs) as regulators of inflammatory events associated with thyroid pathology [4-6]. Interestingly, in animal models of spontaneous thyroiditis an accumulation of DCs in thyroid has been shown prior to autoantibody occurrence and clinical signs [7], suggesting their engagement in the earliest phases of pathological events. The extraordinary regulatory properties of DCs depend strongly on their maturity and activation state and differ considerably between particular DCs subtypes of which the most important in human settings are myeloid and plasmacytoid DCs (mDCs and pDCs, respectively) [8]. Recently we have shown for the first time a regulatory action of thyroid hormones (TH) on naturally occurring human peripheral blood DCs [9]. For that purpose we compared *ex vivo* the phenotype and immunoregulatory properties of peripheral blood DCs subtypes in thyroidectomized patients in two thyrometabolic states – during withdrawal of L-thyroxine (L-T4) treatment and, subsequently, after administration of L-T4 for 2–3 months. In this experimental model, we observed that the supplementation of L-T4 resulted in a significant quantitative increase of plasmacytoid and myeloid DCs circulating in patients’ peripheral blood, as well as in an up-regulation of the surface expression of CD86 co-stimulatory molecule (regarded as the main DCs maturation marker) on both DCs subtypes. Similar effects of TH supplementation were also observed in culture experiments with magnetically sorted human peripheral blood DCs. Although the results of *in vitro* experiments confirmed the direct influence of TH on DCs, the role of TSH fluctuations *in vivo* in the immunological observations associated with TH treatment could not be fully excluded [9].

The aim of this study was to further investigate the influence of TSH on the structure and co-stimulatory phenotype of human peripheral blood DCs subtypes *ex vivo*. The study was performed in patients thyroidectomized because of differentiated thyroid carcinoma and qualified for recombinant human TSH (rhTSH) administration from standard indications. This unique clinical model, encompassing cytometric analysis of peripheral blood obtained before and after rhTSH administration, allowed the *ex vivo* assessment of TSH influence on human DCs subtypes under stable TH concentrations.

## Methods

### Patients

The study participants were recruited from the Department of Endocrinology and Metabolic Diseases, Polish Mother’s Memorial Hospital – Research Institute in Lodz. Prior to the enrolment, all of the participants signed an informed consent, according to the study protocol approved by the local Ethics Committee. The study group included 24 patients (19 women and 5 men, age 50,6 ± 12,75 years, mean ± SD) thyroidectomized because of differentiated thyroid carcinoma (papillary thyroid carcinoma, n = 22 or follicular thyroid carcinoma, n = 2). Total thyroidectomies were performed earlier [mean time period before our study 7,17 ± 5,2 years (mean ± SD; range: 1–20 years)]. The patients with metastases, immunological or metabolic disorders (i.e. diabetes mellitus), as well as patients with clinical or laboratory signs of ongoing inflammatory processes were excluded from the study. At the time of study participation, the patients received rhTSH (Thyrogen, Genzyme Corporation; 0.9 mg i.m., followed by second 0.9 mg i.m. injection 24 hours later) as a routine control of potential thyroid cancer activity.

Peripheral blood samples were collected between 08.00 and 09.00 AM after an overnight fast. Venous blood was obtained by clean venipuncture (needle gauge 19), avoiding slow flowing draws and/or traumatic venipunctures. The blood samples were collected from the same patient (n = 24) at two (2) consecutive time points: (i) directly before the commencement of rhTSH administration and (ii) five (5) days after first rhTSH injection.

Free triiodothyronine (FT3), free thyroxine (FT4) and TSH concentrations were measured by the immunoradiometric (IRMA) method with appropriate kits (BRAHMS, Berlin, Germany; range normal values: TSH: 0.27–4.2 mIU/L; FT3: 2,6–4,4 pg/mL; FT4: 0,93–1,7 ng/dL). The concentration of thyroglobulin (Tg) was assessed with Elecsys Tg reagent kit and Cobas e 411 analyser (Roche Diagnostic Mannheim, Germany) and the concentration of Tg antibody was measured by the electrochemiluminescence (ECLIA) method with appropriate kits (Roche Diagnostic Mannheim, Germany, normal value: Tg antibody & 115 IU/ml) and equipment (Modular Analytics E170 - Roche Diagnostic).

### Fluorescence-activated cell sorting (FACS) analysis

Whole blood samples obtained from study participants were assessed on the same day by flow cytometry, using a FACSCanto II® cytometer and FACSDiva® software (BD Biosciences, San Jose, CA, USA). Peripheral blood DCs subsets can be recognized on the basis of surface expression pattern of blood dendritic cell antigens (BDCAs). Two distinct populations of mDCs are characterized by expression of: BDCA1 (which has been shown to be identical to CD1c) – mDC1, or CD141/BDCA3 – mDC2. In contrast, BDCA2 and BDCA4 are specific for blood pDCs [10-12]. Monoclonal antibodies (mAb) specific for BDCA antigens were purchased from Miltenyi Biotec (Bergisch Gladbach, Germany). All remaining mAb and appropriate isotype controls were purchased from BD Biosciences Pharmingen (San Jose, CA, USA). The staining of peripheral blood DCs subtypes was performed as described earlier [13,14]. Shortly, the BDCA2+ plasmacytoid DCs and the BDCA3+ myeloid DCs subtypes were recognized by staining with allophycocyanin (APC) conjugated antibodies specific for BDCA2 (AC144, mouse IgG1), and BDCA3 (AD5-14H12, mouse IgG1), respectively. The CD1c/BDCA1+ subtype was recognized as a population positive for anti-BDCA1-APC (AD5-8E7, mouse IgG2a) and negative for anti-CD19-PerCP-Cy5.5 (HIB19, mouse IgG1) staining. All DCs populations were counterstained with phycoerythrin (PE) conjugated mAb: anti-CD11c (B-ly6, mouse IgG1) and anti-CD86 (2331/FUN-1, mouse IgG1). After the incubation with mAb erythrocytes were lysed (15 minutes at room temperature) with FACS Lysing Solution (BD Biosciences, San Jose, CA, USA). The leukocyte fraction was then washed twice with cold phosphate buffered saline (PBS), counted and suspended in PBS for FACS analysis. To avoid an unspecific antibody-binding, a FcR-blocking reagent (Miltenyi Biotec, Bergisch Gladbach, Germany) was applied in all analyses. DCs subpopulations were analyzed in a blinded way and the results were expressed as a percentage of peripheral blood mononuclear cells (PBMC) fraction.

### Statistical analysis

Statistical analysis was performed using the Prism 5.0 statistical software (GraphPad, La Jolla, CA, USA). The normality of distribution was assessed utilizing the Shaphiro-Wilk test and the differences in peripheral blood cell populations were analyzed with the Wilcoxon matched-pairs signed rank test. In all the analyses, results were considered statistically significant when p & 0.05.

## Results

### Clinical characteristics

All the study participants received suppressive L-T4 treatment (mean L-T4 dose: 150 ± 32.8 μg/day ±SD). At the commencement of the study the serum concentration of FT3 was 3,39 ± 0,6 pg/mL (mean ± SD), concentration of FT4 was 1,92 ± 0,4 ng/dL and the serum concentration of TSH was 0,18 ± 0,3 mIU/L (range of normal values: FT3: 2,6–4,4 pg/mL; FT4: 0,93–1,7 ng/dL; TSH: 0.27–4.2 mIU/L). The serum Tg concentration assessed five (5) days after the first rhTSH injection, as an indirect measure of malignant process activity, did not show pathological results in any of the study participants. The concentration of Tg was 2,12 ± 4,7 ng/dL and the concentration of Tg antibody was 28,59 ± 68,4 IU/ml (normal value: Tg antibody & 115 IU/ml).

### The percentage of human peripheral blood DCs subtypes was not influenced by rhTSH administration

**Figure 1:**
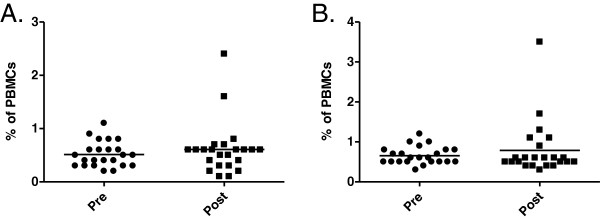
**The influence of TSH on distribution of human peripheral blood DCs subtypes.** The percentage of pDCs (**A**) and CD1c/BDCA+ mDCs (**B**) in the whole PBMC fraction of patients (n = 24) directly before commencement of rhTSH administration (pre) and 5 days after first rhTSH injection (post).

The administration of rhTSH did not influence the percentage of plasmacytoid DCs in peripheral blood of study participants (0.513 ± 0.238% vs. 0.609 ± 0.495%; p = 0.41). Also the percentage of the main myeloid DCs population – CD1c/BDCA1+ mDCs did not change significantly after rhTSH injections (0.657 ± 0.219% vs. 0.787 ± 0.681%; p = 0.88) as compared to the value directly before therapy commencement (Figure 1). In accordance with earlier observations, the percentage of the second myeloid DCs population – CD141/BDCA3+ mDCs was very low in all the patients and did not show any significant quantitative fluctuations during the study (0.1 ± 0.02% vs. 0.1 ± 0.03%; p = 1.0).

### Administration of rhTSH did not change maturation state of human peripheral blood DCs subtypes

**Figure 2:**
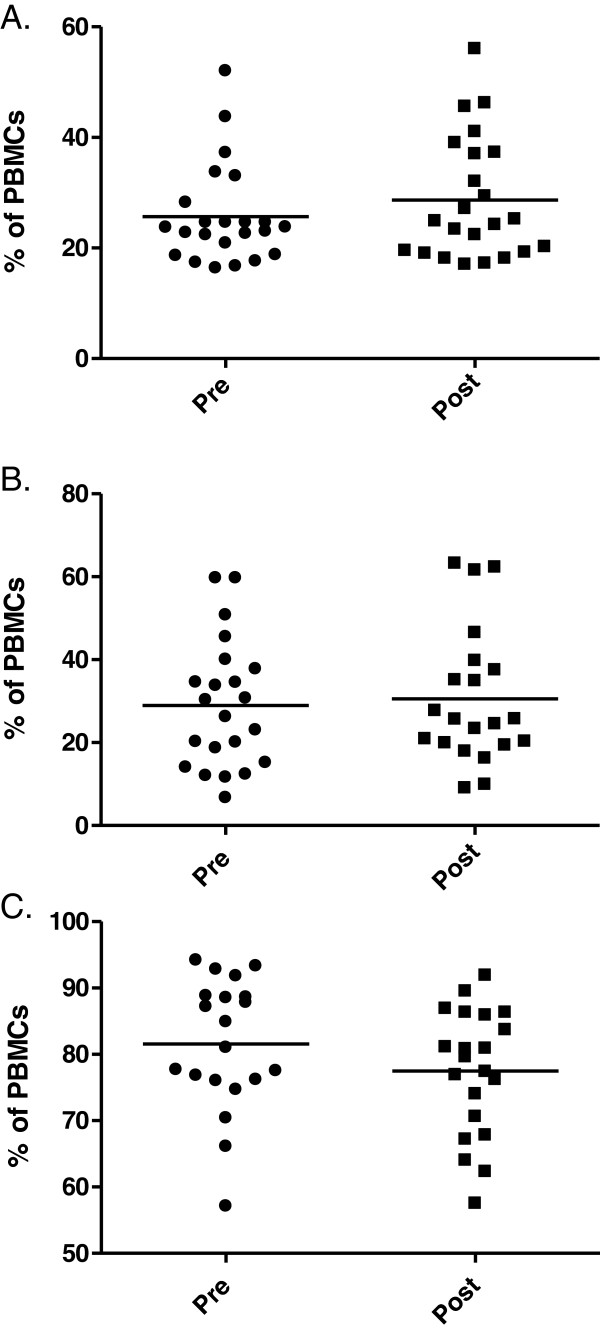
**The influence of TSH on CD86 expression in peripheral blood.** The precentage of CD86 positive cells (**A**), pDCs (**B**) and CD1c/BDCA+ mDCs (**C**) in the whole PBMC fraction of patients (n = 24) directly before commencement of rhTSH administration (pre) and 5 days after first rhTSH injection (post).

In order to better characterize the potential effect of TSH on human DCs subtypes, we performed an assessment of surface expression of CD86 – one of the major co-stimulatory molecules, regarded as DCs maturation marker. In our analysis the administration of rhTSH did not influence the level of CD86 expression in the whole PBMC population (Figure 2A). Also the percentage of CD86 positive pDCs and CD1c/BDCA1+ mDCs did not change after rhTSH injection (28.98 ± 15.44% vs. 30.54 ± 16.28%; p = 0.53 and 81.57 ± 9.96% vs. 77.47 ± 9.54%; p = 0.08, respectively) (Figure 2B and C). Due to the very low numbers of CD141/BDCA3+ mDCs the expression of CD86 could not be analyzed statistically in this population.

## Discussion

Numerous hormonal factors including androgens, estrogens, progesterone, glucocorticosteroids and vitamin D have been shown to modulate the immune properties of DCs of both myeloid and plasmacytoid characteristics [15-18]. However, very little is known about the influence of hypothalamus-pituitary-thyroid axis on DCs subtypes and their biological function. Lately, expression of TH receptor was reported in murine bone marrow derived DCs. The *in vitro* experiments showed that T3 stimulation resulted in TH-receptor depended activation of NF-κB and Akt transcriptional pathways and influenced maturation and immune function of these cells [19,20]. Interestingly, the direct immunostimulatory effect of T3 on murine DCs could be prevented by glucocorticosteroids in mechanism involving down-regulation of TH-receptor expression, as well as inhibition of Akt and NF-κB signaling [21]. In our recent work, we have demonstrated similar stimulatory influence of TH on human peripheral blood DCs both *ex vivo* and *in vitro*. Although the *in vitro* experiments confirmed the direct nature of TH action on human DCs, we could not fully exclude that the immunomodulatory effects observed *ex vivo*, were partially dependent on serum TSH fluctuations associated with L-T4 treatment [9]. TSH receptor expression was described earlier on murine DCs and TSH stimulation was shown to augment the phagocytic properties and secretion of proinflammatory cytokines (IL1-beta and IL-12) by DCs [22]. In human settings, expression of TSH receptor was presented most recently in pluripotent bone marrow mesenchymal stem cells [23] but there is little knowledge about TSH receptor expression in hematopoietic lineages and main human leukocyte subsets. In our present work we have performed for the first time an analysis of the influence of TSH on human peripheral blood DCs subsets *ex vivo*. The unique clinical model, based on the thyroidectomized patients receiving systemically rhTSH, provided the most natural, independent from TH fluctuations, experimental conditions for such assessment in humans. In contrast to the results of the aforementioned animal study *in vitro*, in our experiments we did not observe any significant influence of TSH on human peripheral blood DCs *ex vivo*. Systemic administration of rhTSH did not cause any significant quantitative changes in the assessed plasmacytoid and myeloid DCs subpopulations. Also the level of CD86 surface expression on particular DCs subsets was not influenced by rhTSH. Beside the obvious species differences, the lack of effect of systemic rhTSH administration on peripheral blood DCs observed in our study may suggest that the TSH-DCs interaction reaches its significance rather on the level of local, organ-specific, regulatory circuits. This suggestion stays in accordance with results of experimental studies performed on DCs isolated from the thyroid gland. It was shown that the maturation state and proliferative response of thyroid derived DCs co-cultured *in vitro* with thyrocytes depended strongly on TSH stimulation and humoral factors secreted by thyrocytes (GM-SCF and TGF-beta) [24,25]. The dominant role of auto- and paracrine TSH stimulation in DCs biology seems to be also supported by observations that DCs represent an effective local source of TSH under physiological and inflammatory conditions [26,27].

## Conclusions

Results of this study are in concordance with the direct, independent from TSH, effect of TH on human peripheral blood DC subtypes suggested by the results of our previous experiments *ex vivo*. This observation extends the knowledge about the regulatory processes of human DCs function and may be of great importance in research directed on DCs based therapeutic approaches.

## Abbreviations

BDCA: Blood dendritic cell antigen; DCs: Dendritic cells; FACS: Fluorescence-activated cell sorting; FT3: Free triiodothyronine; FT4: Free thyroxine; L-T4: L-thyroxine; mDCs: Myeloid dendritic cells; pDCs: Plasmacytoid dendritic cells; PBMC: Peripheral blood mononuclear cell; TH: Thyroid hormones; Tg: Thyroglobulin; TSH: Thyrotropin; rhTSH: Recombinant human thyrotropin.

## Competing interests

The authors declare that no competing financial interests exist.

## Authors’ contributions

MS carried out the flow cytometry analysis and drafted the manuscript. ZA participated in the study design, carried out the clinical examination and patient recruitment. BP participated in the design of the study and performed the statistical analysis. KKR participated in the patient recruitment and performed the analysis of clinical parameters. AZ participated in the patient recruitment and performed the analysis of clinical parameters. MB participated in the analysis of flow cytometry data. PD participated in the analysis of the results and helped to draft the manuscript. AL conceived of the study, and participated in its design and coordination and prepared the final version of the manuscript. All authors read and approved the final manuscript.
